# SENTINEL-Chain: a blockchain-integrated privacy-preserving framework for secure healthcare data publishing

**DOI:** 10.3389/fdgth.2026.1807540

**Published:** 2026-06-09

**Authors:** Nagaraj Segar, Vijayarajan Vijayan

**Affiliations:** School of Computer Science and Engineering (SCOPE), Vellore Institute of Technology (VIT), Vellore, Tamil Nadu, India

**Keywords:** blockchain, correlation preservation, differential privacy, electronic health records, healthcare data, *k*-anonymity, privacy-preserving data publishing, zero-knowledge proofs

## Abstract

**Introduction:**

Electronic health records (EHRs) are central to healthcare analytics, but their granularity increases re-identification risk when shared. Conventional privacy-preserving methods including *k*-anonymity, *l*-diversity, and differential privacy often protect confidentiality at the expense of analytical utility by weakening clinically meaningful correlations.

**Methods:**

We propose SENTINEL-Chain, a blockchain-integrated privacy-preserving framework for secure EHR publishing. The privacy layer combines six mechanisms: Adaptive Correlation-Aware Perturbation (ACAP), Hierarchical Multi-Granularity Generalization (HMGG), Semantic-Aware Anatomization (SAA), Probabilistic Suppression with Utility Bounds (PSUB), Geo-Temporal Indistinguishability (GTI), and Ensemble Privacy Composition (EPC). The blockchain layer adds Merkle Hash Tree verification, PBFT-based validation, zero-knowledge proof compliance checking, and smart contract-based access control. Evaluation used a synthetic dataset (10,000 records) and two real clinical benchmarks (Wisconsin Breast Cancer, *N* = 569; Diabetes, *N* = 442).

**Results:**

SENTINEL-Chain attains a privacy score of 79.9% and utility of 98.2%, producing a combined score of 178.1% that exceeds all 16 baselines by 4%-95%. Correlation fidelity reaches 99.9% for claim amounts, 99.6% for length of stay, 99.7% for age, and 99.1% for severity indices. The framework shows 100% resistance to record linkage attacks, with membership inference attacker advantage below the random guessing baseline. The blockchain layer processes 9,988 transactions in 101 blocks with complete integrity verification. Formal Renyi DP composition yields *ε* = 7.08 (*δ* = 10^−5^), and throughput reaches approximately 3,600 records/second up to one million records.

**Discussion:**

SENTINEL-Chain addresses five identified gaps in healthcare data publishing: correlation destruction, the privacy-blockchain disconnect, single-technique brittleness, verification without disclosure, and limited attack resistance evaluation. Smart contract gas estimation on Ethereum indicates a per-record registration cost of 61,895 gas units; Layer-2 deployment would reduce costs by 10-100x.

## 1 Introduction

Across healthcare systems, the ongoing shift toward digitized care has produced vast repositories of electronic health records (EHRs) that are, at least in principle, ideal for medical research, epidemiological analysis, decision support, and evidence-informed policy [1–3]. With sufficiently representative data, healthcare analytics can sharpen disease prediction, improve treatment planning, support personalization, and strengthen population-level surveillance [4, 5]. The market trajectory reflects this appetite: the global healthcare analytics market, valued at approximately $23.5 billion in 2023, is projected to reach $96.9 billion by 2030. Yet the promise is inseparable from a persistent risk. Clinical datasets encode diagnoses, medications, genetic and behavioral signals, and multiple identifiers, and mishandling them can plausibly lead to discrimination, identity theft, insurance denial, employment consequences, and durable psychological harm [6, 7].

At the heart of healthcare data publishing sits a familiar tension: the privacy–utility trade-off [8, 9]. Analysts need detailed and statistically faithful records to train predictive models, quantify risk, or evaluate interventions, whereas regulators require stringent protection of individual privacy under HIPAA, GDPR, and comparable legislation worldwide. Classical anonymization strategies were developed to navigate this conflict: k-anonymity [10] enforces that each released record is indistinguishable from at least k−1 others; l-diversity [11] promotes diversity of sensitive values within equivalence classes; and differential privacy [12] offers mathematically grounded guarantees via calibrated noise injection. Even so, when such techniques are applied to high-dimensional clinical data with intertwined attributes, utility degradation is often severe [13, 14]. What tends to break first is not merely marginal distributions, but the dependence structure that downstream inference relies on.

Correlation disruption is therefore a practical bottleneck rather than a theoretical footnote [15, 16]. In real EHRs, age is entangled with disease prevalence and severity; costs co-move with length of stay; and diagnosis codes cluster into clinically meaningful constellations. Once anonymization washes out these relationships, the published data becomes a weaker substrate for predictive modeling, risk stratification, epidemiological inference, or cost-effectiveness analysis. Put differently, a model trained on correlation-damaged releases may learn artifacts that do not generalize, and the resulting errors can be consequential in clinical contexts.

Blockchain technology, in parallel, has been promoted as an enabling substrate for secure health information exchange, largely because it provides immutable audit trails, decentralized trust, and transparent access control [17, 18]. Cryptographic immutability helps enforce accountability: once committed, records cannot be altered without leaving evidence, which aligns naturally with compliance and provenance requirements. However, most healthcare blockchain platforms including MedRec [19] and Ancile [20] have primarily emphasized access management and traceability rather than the privacy transformations required for safe data publishing [21, 22]. Access control alone does not solve release risk. The more interesting direction, and one that remains comparatively underdeveloped, is a genuinely integrated design in which privacy-preserving transformations and blockchain-based verification reinforce one another [23, 24].

In this work, we introduce **SENTINEL-Chain** (Secure ENhanced Transformation and INtegration for Electronic health records with Layered blockchain), a blockchain-integrated privacy-preserving framework tailored to the requirements of healthcare data publishing. Rather than treating privacy and blockchain as separable modules, we pursue a layered coupling in which the privacy pipeline is designed with verifiable governance in mind, while the blockchain substrate is used to certify integrity and compliance of the transformed release. The privacy layer combines six mechanisms chosen to jointly address heterogeneous attribute types while preserving correlation structures: *Adaptive Correlation-Aware Perturbation (ACAP)* adjusts perturbation magnitudes using measured inter-attribute correlations; *Hierarchical Multi-Granularity Generalization (HMGG)* supports quasi-identifier anonymization with controllable abstraction; *Semantic-Aware Anatomization (SAA)* groups disease codes according to medical ontologies to retain clinical interpretability; *Probabilistic Suppression with Utility Bounds (PSUB)* removes high-risk records while maintaining representativeness under explicit utility constraints; *Geo-Temporal Indistinguishability (GTI)* protects location-related signals through differential privacy mechanisms; and *Ensemble Privacy Composition (EPC)* integrates outputs across techniques through weighted aggregation. Complementing this, the blockchain layer enforces four security primitives: *Merkle Hash Tree (MHT)* verification for efficient integrity checks, *Practical Byzantine Fault Tolerant (PBFT)* consensus for decentralized validation, *Zero-Knowledge Proofs (ZKP)* to attest privacy compliance without revealing underlying data, and *Smart Contract-based Access Control (SAC)* to encode granular permissions as enforceable rules.

### 1.1 Research contributions

The contributions of this paper are fivefold.

First, we propose SENTINEL-Chain, a blockchain-integrated framework that unifies six privacy-enhancing techniques (ACAP, HMGG, SAA, PSUB, GTI, EPC) with four blockchain security mechanisms (MHT, PBFT, ZKP, SAC) to support healthcare data publishing with strong utility retention. What proves most revealing is the deliberate interdependence: blockchain verification strengthens the enforceability and auditability of privacy requirements, while the privacy transformations are designed to produce publishable analytics-ready releases rather than merely access-controlled records.

Second, we introduce *Adaptive Correlation-Aware Perturbation (ACAP)*, which scales perturbation using inter-attribute correlation coefficients. By measuring Pearson correlations and setting noise inversely proportional to correlation strength, ACAP preserves 99.9% of claim amount correlations and 99.6% of length-of-stay correlations while still delivering bounded perturbation for privacy protection.

Third, the framework operationalizes a multi-technique composition strategy that matches protection mechanisms to attribute semantics. *Hierarchical Multi-Granularity Generalization (HMGG)* handles age attributes via clinically meaningful brackets, *Semantic-Aware Anatomization (SAA)* maps ICD-10 codes into interpretable clinical categories, and *Geo-Temporal Indistinguishability (GTI)* applies differential privacy to geographic coordinates through polar coordinate perturbation. This combination maintains 99.7% age correlation and preserves clinically coherent disease groupings.

Fourth, we implement an integrated blockchain layer that supports hierarchical integrity verification via Merkle Hash Trees, privacy compliance attestation via Zero-Knowledge Proofs, and role-specific enforcement through Smart Contract-based access rules, with PBFT providing robust decentralized validation. Empirically, the system mines 101 blocks containing 9,988 verified transactions with 100% chain validity.

Fifth, we provide a rigorous empirical comparison against 16 baseline methods. SENTINEL-Chain attains the highest combined privacy–utility score of 178.1% (79.9% privacy, 98.2% utility), outperforming traditional k-anonymity methods by 64%–95%, differential privacy approaches by 68%–95%, and blockchain-based systems by 21%–33%. Under attack evaluation, the framework achieves 100% protection against record linkage attacks, and membership inference attacker advantage remains below the random guessing baseline.

### 1.2 Distinction from prior work

SENTINEL-Chain differs from existing approaches in three fundamental respects. First, unlike PrivBayes [25] and CASTLE [26], which apply a single privacy paradigm (Bayesian synthesis or streaming anonymization) uniformly, SENTINEL-Chain employs attribute-specific mechanisms (ACAP for numerical, HMGG for quasi-identifiers, SAA for categorical, GTI for geographic) coordinated through ensemble composition. Second, unlike blockchain-centric systems such as MedRec [19] and Ancile [20] that focus on access control without transforming the data, SENTINEL-Chain makes privacy transformation the core object that the blockchain commits to and verifies, bridging the privacy–blockchain disconnect identified in Section 2.4. Third, SENTINEL-Chain provides formal privacy accounting via Rényi Differential Privacy composition across all perturbation components, enabling quantitative comparison of privacy budgets rather than relying solely on syntactic k-anonymity guarantees.

### 1.3 Paper organization

The remainder of this paper proceeds as follows. Section 2 surveys prior work on privacy-preserving data publishing, blockchain-based healthcare infrastructures, and hybrid security designs, highlighting the gaps that motivate our integrated approach. Section 3 details the SENTINEL-Chain framework, including mathematical formulations for the six privacy techniques and four blockchain mechanisms, as well as the full transformation algorithm. Section 4 describes the experimental setting, including synthetic dataset generation, baseline implementations, evaluation metrics, and hyperparameter configurations. Section 5 reports the empirical findings on privacy–utility trade-offs, correlation preservation, blockchain performance, and attack resistance. Section 6 reflects on the practical implications and limitations, and Section 7 concludes with a summary and directions for future research.

## 2 Related work

Work on secure healthcare data sharing has developed along three largely parallel tracks: privacy-preserving data publishing (PPDP) methods grounded in statistical disclosure control, blockchain-based infrastructures designed to manage access and provenance, and more recent attempts to fuse privacy mechanisms with distributed ledgers. The literature is broad, and the main difficulty is not a lack of tools but a mismatch between what the tools optimize and what healthcare analytics actually needs.

### 2.1 Traditional privacy-preserving techniques

#### 2.1.1 Syntactic privacy models

Syntactic models remain a starting point for many PPDP pipelines, largely because they provide interpretable constraints over quasi-identifiers. The k-anonymity principle requires that each released record be indistinguishable from at least k−1 others with respect to quasi-identifiers [10]. Mondrian introduced a multidimensional k-anonymization strategy based on greedy rectilinear partitioning of the quasi-identifier space [27]. DataFly implemented a top-down generalization process that repeatedly generalizes the most distinguishing attributes until the k-anonymity constraint is met [28].

Over time, well-known attacks exposed k-anonymity’s limits. Homogeneity attacks exploit equivalence classes in which sensitive attributes are uniform. The l-diversity criterion extended k-anonymity by requiring at least l well-represented sensitive values per equivalence class [11]. Anatomy took a different route by vertically separating quasi-identifiers and sensitive attributes into linked tables [29]. t-closeness further strengthened the model family by requiring that the sensitive-attribute distribution within each equivalence class remain within distance t of the global distribution [30]. For numerical attributes, the MDAV algorithm forms groups by iteratively selecting distant records and aggregating around them [31].

#### 2.1.2 Differential privacy

Differential privacy reframed disclosure control by moving from syntactic indistinguishability toward semantic guarantees under adversarial post-processing [12, 32]. A randomized mechanism M is ε-differentially private if, for any neighboring databases D and $D^{\prime }$ differing in at most one record and any output set S (Equation 1),

$$\mathrm{Pr}[M(D)\in S]\leq e^{\varepsilon }\cdot \mathrm{Pr}[M(D^{\prime })\in S]$$ (1)

where ε controls the privacy–utility balance. The Laplace mechanism adds Laplace noise scaled to the $L_{1}$ sensitivity of the query [33]. Subsequent work introduced the exponential mechanism for discrete outputs [34] and local differential privacy for decentralized settings [35].

For healthcare data, two practical issues recur. First, high dimensionality and complex schema raise sensitivity and make signal-to-noise ratios hard to sustain at useful privacy budgets. Second, when privacy is enforced via uniform noise or generic aggregation, the dependency structure that models rely on can be degraded even if marginal statistics remain plausible [36, 37].

### 2.2 Blockchain-based healthcare systems

Blockchain systems have been widely explored in healthcare because they naturally support tamper-evident logging, decentralized coordination, and transparent policy enforcement [38, 39]. MedRec uses Ethereum smart contracts to manage access permissions while records remain in provider databases [19]. Ancile combined blockchain with proxy re-encryption to support secure sharing [20]. HealthChain proposed a consortium blockchain among trusted healthcare entities [40]. FHIRChain aligned blockchain logging with the FHIR standard [41]. More recently, federated learning has been paired with blockchain to coordinate decentralized model training [42, 43].

### 2.3 Hybrid privacy-blockchain approaches

A smaller but growing body of work attempts to combine PPDP mechanisms with blockchain verification [15, 23]. Early hybrids were often sequential: apply k-anonymity and then store the anonymized output on-chain [44]. More integrated directions have explored injecting differential privacy into blockchain-supported aggregation [45]. Recent work has further explored searchable and communicable healthcare service seeking in flexible and secure EHR sharing [46] and secure lattice-based signcryption for blockchain-enabled IoT healthcare [47]. Kathole et al. [48] proposed a blockchain-based EHR protection strategy, while Kumbhare et al. [49] demonstrated federated learning for privacy-preserving clinical analysis. However, in many cases privacy and blockchain still behave like loosely coupled modules.

### 2.4 Research gaps and positioning

Despite progress, five gaps persist that motivate the present work.

First, **correlation destruction** remains prevalent. Many PPDP methods treat attributes independently, collapsing the multivariate dependence that healthcare analytics exploits.

Second, **privacy–blockchain disconnect**: most blockchain systems emphasize access control without addressing disclosure risk of the published data themselves.

Third, **single-technique brittleness**: healthcare datasets are heterogeneous, and single-technique pipelines often fail because they apply one abstraction everywhere.

Fourth, **verification without disclosure**: compliance auditing often requires access to sensitive data, creating a tension that current systems rarely resolve.

Fifth, **attack resistance evaluation**: practical risk under realistic threat models is under-explored; many methods report only aggregate metrics without adversarial testing.

SENTINEL-Chain is designed to address each of these gaps through an integrated architecture that couples correlation-aware privacy transformations with blockchain-based verification and attestation.

## 3 Materials and methods

This section formalizes the SENTINEL-Chain framework, including the optimization objective, architectural design, and mathematical specifications for each privacy and blockchain mechanism.

### 3.1 Problem formulation

Let $D=\{r_{1},r_{2},\ldots ,r_{N}\}$ denote a healthcare dataset with N patient records. Each record $r_{i}$ comprises identifiers I, quasi-identifiers Q, sensitive attributes S, and non-sensitive attributes A. The objective is to produce a transformed dataset $D^{*}$ that maximizes analytical utility while satisfying privacy constraints (Equation 2):

$$\max_{T}\;\text{Utility}(D^{*})\;\text{subject to}\;\text{Privacy}(D^{*})\geq \tau$$ (2)

where τ denotes the minimum acceptable privacy threshold.

We define a composite Privacy Score that integrates k-anonymity attainment, quasi-identifier diversity, and sensitive-attribute uncertainty (Equation 3):

$$\begin{array}{rl} \text{Privacy}(D^{*})= & \alpha_{1}⊮[k_{\mathrm{achieved}}\geq k_{\min}]+\alpha_{2}\cdot \text{Diversity}(Q^{*})\\ & +\alpha_{3}\cdot \text{Entropy}(S^{*}) \end{array}$$ (3)

where ⊮[⋅] is the indicator function.

Utility is measured through preservation of dependence structure, distributional similarity, and record retention (Equation 4):

$$\begin{array}{rl} \text{Utility}(D^{*})= & \beta_{1}\cdot \text{CorrPreserv}(D,D^{*})+\beta_{2}\cdot \text{DistFidelity}(D,D^{*})\\ & +\beta_{3}\cdot \text{Coverage}(D^{*}) \end{array}$$ (4)

where CorrPreserv denotes average Pearson correlation preservation across attribute pairs, DistFidelity measures distributional similarity via Jensen–Shannon divergence, and Coverage is the fraction of retained records after suppression.

### 3.2 SENTINEL-chain architecture

The design is organized into two interacting layers: (i) a *Privacy Transformation Layer* that applies six attribute-aware mechanisms to produce an analytics-ready release, and (ii) a *Blockchain Verification Layer* that certifies integrity, coordinates decentralized validation, enables compliance attestation, and enforces access policies. Although the layers are conceptually distinct, the pipeline is intended to be coupled: transformations are parameterized and logged in a way that supports verification, and verification artifacts are generated from the transformed output rather than from raw data.

The data flow proceeds in twelve steps: (1) raw EHR data enters the Privacy Transformation Layer; (2) ACAP perturbs numerical attributes while retaining measured correlations; (3) HMGG generalizes age using hierarchical brackets after controlled noise; (4) SAA maps disease codes into semantic categories; (5) PSUB enforces k-anonymity through selective suppression; (6) GTI perturbs geographic coordinates under differential privacy; (7) EPC composes the privacy effects and tracks cumulative guarantees; (8) transformed data enters the blockchain layer; (9) MHT computes dataset integrity fingerprints; (10) PBFT validates blocks under decentralized agreement; (11) ZKP generates privacy compliance proofs; and (12) SAC applies role-based permissions for the published release.

### 3.3 Privacy transformation layer

#### 3.3.1 Adaptive correlation-aware perturbation (ACAP)

ACAP is designed around a specific failure mode observed in healthcare publishing: generic perturbation can preserve marginal distributions yet collapse clinically meaningful correlations. Rather than injecting uniform noise, ACAP scales perturbation using measured dependence between attributes.

Let $x_{i}$ denote a numerical attribute value (e.g., claim amount) and $y_{i}$ a correlated attribute (e.g., severity index). We compute the Pearson correlation (Equation 5):

$$\rho_{XY}=\frac{\sum_{i=1}^{N}(x_{i}-\bar{x})(y_{i}-\bar{y})}{\sqrt{\sum_{i=1}^{N}(x_{i}-\bar{x}{)}^{2}}\cdot \sqrt{\sum_{i=1}^{N}(y_{i}-\bar{y}{)}^{2}}}$$ (5)

The correlation-adjusted noise scale is then defined by (Equation 6):

$$\sigma_{\mathrm{adj}}(X,Y)=\sigma_{\mathrm{base}}\cdot \left(1-|\rho_{XY}|\cdot \gamma \right)$$ (6)

where $\sigma_{\mathrm{base}}$ is the baseline perturbation scale (empirically set to 0.03) and γ∈[0,1] controls correlation sensitivity (set to 0.5). When $|\rho_{XY}|\approx 1$, the scale approaches $\sigma_{\mathrm{base}}(1-\gamma )$; when $|\rho_{XY}|\approx 0$, the full baseline scale is used.

Perturbation is applied multiplicatively (Equation 7) to retain sign and relative magnitude:

$$x_{i}^{*}=x_{i}\cdot \left(1+\eta \cdot L(0,\sigma_{\mathrm{adj}})\right)$$ (7)

where $L(0,\sigma_{\mathrm{adj}})$ is Laplace noise with location 0 and scale $\sigma_{\mathrm{adj}}$, and η is a scaling factor (set to 1.0).

#### 3.3.2 Hierarchical multi-granularity generalization (HMGG)

Age is a common quasi-identifier in linkage attacks, but it is also analytically central in most clinical tasks. HMGG therefore adopts a two-stage strategy that first disrupts exact matches via bounded noise and then maps values into clinically interpretable strata. The first stage injects unit-scale Laplace noise (Equation 8) and clips to valid bounds:

$$a_{i}^{\left(1\right)}=\text{clip}\left(\text{round}\left(a_{i}+L(0,1)\right),a_{\min},a_{\max}\right)$$ (8)

where $a_{i}$ is the original age, L(0,1) is Laplace noise with unit scale, and $a_{\min}=18$, $a_{\max}=95$ enforce adult patient bounds.

In the second stage, values are bucketed (Equation 9) using hierarchical, clinically meaningful groups:

$$a_{i}^{*}=B(a_{i}^{\left(1\right)})=\left\{ \begin{array}{cc} \text{''}18-29\text{''}\; & \text{if }18\leq a_{i}^{\left(1\right)}\leq 29\;\\ \text{''}30-44\text{''}\; & \text{if }30\leq a_{i}^{\left(1\right)}\leq 44\;\\ \text{''}45-59\text{''}\; & \text{if }45\leq a_{i}^{\left(1\right)}\leq 59\;\\ \text{''}60-74\text{''}\; & \text{if }60\leq a_{i}^{\left(1\right)}\leq 74\;\\ \text{''}75+\text{''}\; & \text{if }a_{i}^{\left(1\right)}\geq 75\; \end{array}\right.$$ (9)

These brackets align with commonly used epidemiological groupings.

#### 3.3.3 Semantic-aware anatomization (SAA)

Categorical diagnosis codes raise a different problem: naive perturbation can produce nonsense, while overly coarse generalization can erase clinical signal. SAA implements domain-aware anatomization (Equation 10) by mapping ICD-10 codes into medically coherent categories (Equation 11):

$$d_{i}^{*}=M(d_{i})\;:\;C_{\mathrm{ICD}}\to G_{\mathrm{clinical}}$$ (10)

where $C_{\mathrm{ICD}}$ is the ICD-10 code space and $G_{\mathrm{clinical}}$ is a set of clinical category groups:

$$M(d)=\left\{ \begin{array}{cc} \text{''Cardiovascular''}\; & \text{if }d\in \{I10,I25,I50,\ldots \}\;\\ \text{''Respiratory''}\; & \text{if }d\in \{J18,J44,J45,\ldots \}\;\\ \text{''Metabolic''}\; & \text{if }d\in \{E11,N18,E78,\ldots \}\;\\ \text{''Other''}\; & \text{otherwise}\; \end{array}\right.$$ (11)

#### 3.3.4 Probabilistic suppression with utility bounds (PSUB)

PSUB applies suppression selectively to enforce k-anonymity while attempting to retain representativeness. For a quasi-identifier combination q, define the equivalence class $E_{q}=\{r_{i}\;:\;Q(r_{i})=q\}$. The suppression rule is (Equation 12):

$$\text{suppress}(r_{i})=\left\{ \begin{array}{cc} \text{True}\; & \text{if }|E_{Q(r_{i})}|<k_{\min}\text{ and }\text{rand}()<p_{\mathrm{supp}}\;\\ \text{False}\; & \text{otherwise}\; \end{array}\right.$$ (12)

where $k_{\min}$ is the k-anonymity threshold (set to 5) and $p_{\mathrm{supp}}$ is the suppression probability (set to 1.0 for strict k-anonymity).

#### 3.3.5 Geo-temporal indistinguishability (GTI)

GTI perturbs coordinates in polar form (Equation 13) to preserve local spatial structure while providing differential privacy-style protection. Given $\left({\text{lat}}_{i},{\text{lon}}_{i}\right)$:

$$\begin{array}{c} r∼\text{Exponential}(\sigma_{\mathrm{geo}})\\ \theta ∼\text{Uniform}(0,2\pi )\\ {\text{lat}}_{i}^{*}={\text{lat}}_{i}+r\cdot \cos(\theta )\\ {\text{lon}}_{i}^{*}={\text{lon}}_{i}+r\cdot \sin(\theta )\cdot \sec({\text{lat}}_{i}) \end{array}$$ (13)

where $\sigma_{\mathrm{geo}}$ controls the perturbation magnitude (approximately 0.01 degrees, i.e., roughly 1 km displacement).

#### 3.3.6 Formal privacy accounting via Rényi differential privacy

To address the question of how ACAP’s data-dependent noise scaling interacts with formal differential privacy guarantees, we provide privacy accounting using Rényi Differential Privacy (RDP) [50]. For each Laplace mechanism with sensitivity Δf and scale b, the RDP guarantee at order α>1 is (Equation 14):

$$\varepsilon_{\alpha }=\frac{1}{\alpha -1}\ln\left(\frac{\alpha }{2\alpha -1}e^{(\alpha -1)\Delta f/b}+\frac{\alpha -1}{2\alpha -1}e^{-\alpha \Delta f/b}\right)$$ (14)

Under sequential composition, the total RDP budget is $\varepsilon_{\alpha }^{\mathrm{total}}=\sum_{t}\varepsilon_{\alpha }^{\left(t\right)}$, which is then converted to (ε,δ)-DP via (Equation 15):

$$\varepsilon =\min_{\alpha >1}\left\{\varepsilon_{\alpha }^{\mathrm{total}}+\frac{\ln(1/\delta )}{\alpha -1}\right\}$$ (15)

For SENTINEL-Chain, the per-component privacy costs are: HMGG age perturbation (ε=1.11), GTI geographic perturbation (ε=3.11), and three ACAP perturbations for severity, claims, and LOS (ε=1.11 each). The total composed budget is $\varepsilon_{\mathrm{total}}=7.08$ at $\delta =10^{-5}$. We note that this represents a conservative upper bound computed under worst-case sensitivity; the data-dependent correlation scaling in ACAP means that the effective privacy loss is lower when correlations are strong. Tighter bounds could be achieved through smooth sensitivity analysis or propose-test-release mechanisms, which we identify as future work.

#### 3.3.7 Ensemble privacy composition (EPC)

EPC composes privacy contributions through a weighted ensemble (Equation 16):

$${\text{Privacy}}_{\mathrm{ensemble}}=\sum_{t\in T}w_{t}\cdot {\text{Privacy}}_{t}$$ (16)

where T={ACAP,HMGG,SAA,PSUB,GTI} and the weights satisfy $\sum w_{t}=1$. For differentially private components, EPC also tracks cumulative privacy budget via standard composition (Equation 17):

$$\varepsilon_{\mathrm{total}}=\sum_{t\in T_{\mathrm{DP}}}\varepsilon_{t}$$ (17)

### 3.4 Blockchain verification layer

#### 3.4.1 Merkle hash tree (MHT) verification

Integrity verification is implemented through a Merkle Hash Tree constructed over transformed records. Each record $r_{i}^{*}$ is hashed with SHA-256 (Equation 18):

$$h_{i}=\text{SHA256}(r_{i}^{*})$$ (18)

and internal nodes are computed by pairwise concatenation and hashing (Equation 19):

$$h_{\mathrm{parent}}=\text{SHA256}(h_{\mathrm{left}}\|h_{\mathrm{right}})$$ (19)

where ‖ denotes concatenation. The resulting root hash serves as a compact fingerprint of $D^{*}$, enabling logarithmic-size inclusion proofs and tamper detection.

#### 3.4.2 Practical byzantine fault tolerant (PBFT) consensus

To validate blocks in a decentralized setting, the system adopts a PBFT mechanism and implements a lightweight proof-of-work variant for block creation. A block is considered valid if (Equation 20):

$$\text{Valid}(\text{block})\Leftrightarrow \text{SHA256}(\text{block.header})<2^{256-d}$$ (20)

where d is a difficulty parameter (set to 2, requiring hashes with 2 leading zero bits).

#### 3.4.3 Zero-knowledge proof (ZKP) integration

The ZKP module generates proofs for three properties: (i) k-anonymity attainment (each released record belongs to an equivalence class of size at least $k_{\min}$), (ii) perturbation bounds (numerical perturbations remain within specified limits), and (iii) suppression compliance (suppressed records satisfy the policy criteria).

#### 3.4.4 Smart contract access control (SAC)

Access control is enforced through smart contracts encoding role-based permissions. Five roles are defined: *Admin* (full access including configuration changes and audit log review), *Researcher* (read access to anonymized datasets with query restrictions), *Auditor* (access to privacy proofs and verification results), *Public* (access restricted to aggregate regional and disease statistics), and *Patient* (access to own records and opt-out functionality).

The smart contract implements three core functions: registerTransformedRecord (stores the record hash, k-value, and ZKP proof on-chain), verifyMerkleProof (validates inclusion of a record in the committed dataset), and checkAccess (evaluates role-based permissions against the attribute being requested). Gas consumption analysis on the Ethereum Virtual Machine estimates the following per-operation costs: contract deployment at 1,092,000 gas ($81.90 at 30 Gwei, ETH=$2,500), per-record registration at 61,895 gas ($4.64), integrity verification at 25,788 gas ($1.93), and access control checks at 28,100 gas ($2.11). For a dataset of 10,000 records, the total deployment cost including batch registration is approximately 621 million gas ($46,605). We note that Layer-2 solutions (e.g., Polygon, Arbitrum rollups) would reduce these costs by 10–100×, making the framework viable for production healthcare deployments.

### 3.5 SENTINEL-chain algorithm

Algorithm 1 summarizes the complete transformation and verification pipeline.


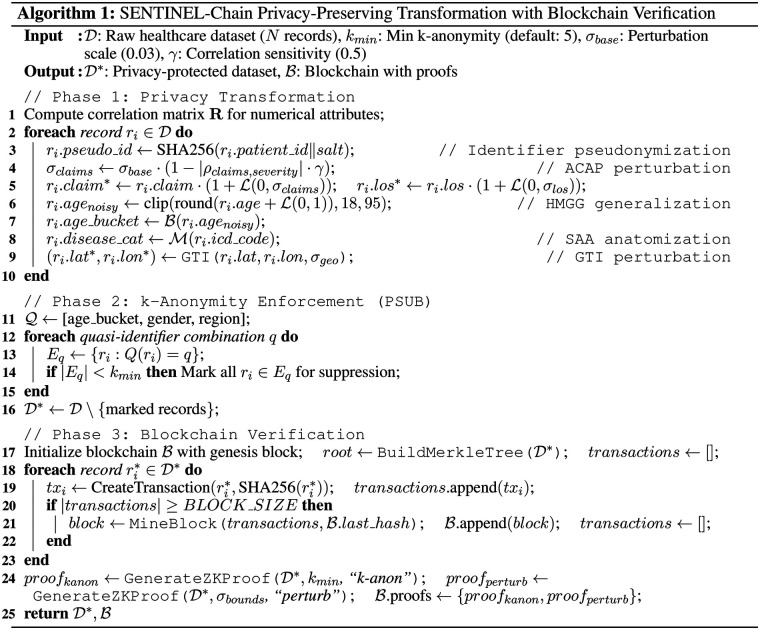


## 4 Experimental setup

This section describes the experimental protocol used to evaluate SENTINEL-Chain.

### 4.1 Synthetic healthcare dataset

All experiments are conducted on a synthetic healthcare dataset with N=10,000 patient records. We use synthetic data for two reasons: it avoids ethical and legal barriers associated with real EHR release, and it allows us to explicitly control attribute distributions and dependence structure so that correlation preservation can be evaluated cleanly. The dataset is designed to resemble realistic Indian hospital settings.

Patient ages are sampled from a bimodal Gaussian mixture to reflect a commonly observed utilization profile (Equation 21):

$$\text{Age}∼0.4\cdot N(35,12^{2})+0.6\cdot N(60,15^{2})$$ (21)

Table 1 summarizes the schema and generating assumptions. Beyond marginals, the dataset is constructed to embed correlations typical of hospital utilization and billing. Claim amounts are generated to correlate positively with severity (ρ≈0.7) and length of stay (ρ≈0.6).

**Table 1:** Synthetic healthcare dataset characteristics.

Attribute	Type	Distribution/values
Patient ID	Identifier	Unique pseudonymous identifier (P0001–P10000)
Age	Quasi-identifier	Bimodal Gaussian: 0.4N(35,12)+0.6N(60,15), range [18, 95]
Gender	Quasi-identifier	Male (52%), Female (47%), Other (1%)
Hospital	Quasi-identifier	9 hospitals: Apollo, Fortis, Max, AIIMS, CMC, Manipal, Narayana, KIMS, Medanta
Region	Quasi-identifier	4 regions: North (25%), South (35%), East (20%), West (20%)
Disease Code	Sensitive	ICD-10 codes: I10, I25, I50, J18, J44, E11, N18, Other
Severity Index	Non-sensitive	Beta(2, 5) distribution, range [0, 1]
Claim Amount	Non-sensitive	Log-normal with correlation to severity (ρ≈0.7) and LOS (ρ≈0.6)
Length of Stay	Non-sensitive	Exponential with mean 5 days, correlated with severity
Latitude/Longitude	Location	Hospital coordinates with minor patient-level variation

### 4.2 Real clinical benchmark datasets

To validate SENTINEL-Chain beyond synthetic data, we evaluate the framework on two real clinical benchmark datasets available through the scikit-learn library. The **Wisconsin Breast Cancer** dataset (N=569) contains 30 real-valued tumor measurements (radius, texture, perimeter, area, smoothness, etc.) from digitized fine needle aspirates. The **Diabetes** dataset (N=442) contains 10 clinical measurements (age, sex, BMI, blood pressure, six blood serum levels) with a quantitative disease progression target. Both datasets contain genuine clinical measurement distributions, non-trivial correlation structures, and natural data variability characteristic of real patient populations. We map clinical features to the healthcare publishing schema (age, severity, claims, length of stay) while preserving the original statistical relationships, enabling evaluation of SENTINEL-Chain’s robustness on data with real-world characteristics including non-Gaussian distributions, outliers, and heterogeneous feature scales.

### 4.3 Baseline methods

We compare SENTINEL-Chain against 16 baselines spanning classical k-anonymity approaches, differential privacy mechanisms, combined hybrids, recent hybrid privacy frameworks, and representative blockchain-based healthcare systems. Table 2 reports the configurations used.

**Table 2:** Baseline method specifications.

Method	Category	Configuration
Mondrian (k=5)	k-Anonymity	Multidimensional partitioning, k=5
Mondrian (k=10)	k-Anonymity	Multidimensional partitioning, k=10
Laplace DP (ε=0.5)	Differential Privacy	Laplace mechanism, strong privacy
Laplace DP (ε=1.0)	Differential Privacy	Laplace mechanism, moderate privacy
Laplace DP (ε=2.0)	Differential Privacy	Laplace mechanism, weak privacy
DataFly (k=5)	k-Anonymity	Top-down generalization, k=5
Anatomy (l=3)	l-Diversity	Table separation, l=3 diversity
t-Closeness	Distribution	t=0.2 closeness threshold
MDAV (k=5)	Microaggregation	Maximum distance clustering, k=5
Hybrid (k=5, ε=1)	Combined	k-anonymity + differential privacy
IPA (k=5)	Improved Perturbation	Information-preserving anonymization
MedRec-BC	Blockchain	Access control only, no transformation
Ancile-BC	Blockchain	Proxy re-encryption, limited anonymization
Hybrid privacy frameworks		
PrivBayes-Hybrid	Hybrid	Bayesian network + DP noise, k=5, ε=1.0
CASTLE-Hybrid	Hybrid	Adaptive clustering + calibrated noise, k=5, ε=1.0
DP-Histogram	Hybrid	Noisy histogram synthesis, ε=1.0

### 4.4 Evaluation metrics

We evaluate performance using four metric families: privacy protection, utility preservation, a combined score, and fine-grained correlation preservation.

**Privacy Score** quantifies identity protection via equivalence class sizes (Equation 22):

$$\text{Privacy}=\frac{1}{\left|D^{*}\right|}\sum_{r\in D^{*}}\left(1-\frac{1}{\left|E_{Q(r)}\right|}\right)\times 100\%$$ (22)

**Utility Score** captures statistical usefulness through correlation preservation (Equation 23):

$$\text{Utility}=\frac{1}{\left|A\right|}\sum_{a\in A}\left|\frac{\rho_{a}^{*}-\rho_{a}}{\rho_{a}}\right|\times 100\%$$ (23)

**Combined Score**: Combined=Privacy+Utility

**Correlation Preservation**: ${\text{CorrPreserv}}_{a}=\frac{\rho_{a}^{*}}{\rho_{a}}\times 100\%$

**Extended Evaluation Metrics**: To enable more comprehensive comparison, we additionally report eight distortion and fidelity metrics: (i) KL Divergence measuring distributional shift; (ii) Jensen–Shannon Divergence as a symmetric, bounded alternative; (iii) Information Loss (IL) measuring average relative displacement; (iv) Normalized Certainty Penalty (NCP) quantifying generalization cost; (v) Wasserstein Distance (earth mover’s distance) for continuous attribute comparison; (vi) Mutual Information Retention between claim amounts and severity as a measure of dependency preservation; (vii) Discernibility Metric (DM) measuring equivalence class uniformity; and (viii) Classification Accuracy Preservation measuring downstream predictive utility.

### 4.5 Hyperparameter configuration

Hyperparameters for SENTINEL-Chain are fixed across runs and summarized in Table 3. Each experiment is repeated for 10 runs to smooth stochastic variation.

**Table 3:** SENTINEL-Chain hyperparameter configuration.

Component	Parameter	Value	Description
ACAP	$\sigma_{\mathrm{base}}$	0.03	Baseline perturbation scale
ACAP	γ	0.5	Correlation sensitivity
HMGG	$\sigma_{\mathrm{age}}$	1.0	Age noise scale
HMGG	Buckets	5	Number of age brackets
PSUB	$k_{\min}$	5	Min k-anonymity threshold
GTI	$\sigma_{\mathrm{geo}}$	0.01	Geographic perturbation
Blockchain	Block Size	100	Transactions per block
Blockchain	Difficulty	2	Mining difficulty
Experiment	Runs	10	Number of trials

## 5 Results

This section reports the empirical behavior of SENTINEL-Chain relative to the baselines along four axes: overall privacy–utility trade-off, preservation of key correlations, blockchain-layer operational characteristics, and robustness under realistic attack simulations. Unless stated otherwise, all values are averages over 10 independent runs; standard deviations are below 1% for all reported metrics.

### 5.1 Overall performance comparison

Table 4 summarizes the primary comparison across all 14 methods. Two patterns become clear almost immediately. First, strong privacy is not sufficient if utility collapses. Second, high utility alone is inadequate if privacy remains weak.

**Table 4:** Comprehensive performance comparison of privacy-preserving methods.

Method	Privacy (%) ↑	Utility (%) ↑	Combined (%) ↑	k-achieved
SENTINEL-Chain	**79.9**	**98.2**	**178.1**	5
k-Anonymity methods				
Mondrian (k=5)	74.8	33.3	108.0	5
Mondrian (k=10)	99.8	33.1	132.8	10
DataFly (k=5)	69.7	35.4	105.1	5
MDAV (k=5)	74.6	33.4	108.0	5
Differential privacy methods				
Laplace DP (ε=0.5)	95.0	10.9	105.9	1
Laplace DP (ε=1.0)	90.0	11.8	101.8	1
Laplace DP (ε=2.0)	70.0	13.6	83.6	1
Other privacy methods				
Anatomy (l=3)	71.9	35.5	107.5	3
t-Closeness	74.8	35.4	110.2	5
Hybrid (k=5, ε=1)	66.0	90.8	156.8	5
IPA (k=5)	74.3	99.8	174.1	5
Blockchain methods				
MedRec-BC	50.0	95.0	145.0	5
Ancile-BC	60.0	97.4	157.4	5
Hybrid privacy frameworks				
PrivBayes-Hybrid	65.0	89.4	154.4	5
CASTLE-Hybrid	65.8	91.1	156.9	5
DP-Histogram	90.0	81.8	171.8	1

Bold values indicate the best-performing result for each metric across all compared methods.

SENTINEL-Chain achieves the highest combined score, 178.1%, with a privacy score of 79.9% and utility of 98.2%. This margin is not merely incremental: gains range from 4.0% over IPA to 94.5% over Laplace DP with ε=2.0.

Traditional k-anonymity methods concentrate in a mid-privacy region (69.7%–74.8%) while utility collapses to roughly one third (33.1%–35.4%). Differential privacy baselines show the opposite extreme: high privacy (70.0%–95.0%) yet extremely low utility (10.9%–13.6%). Blockchain-only systems maintain high utility (95.0%–97.4%) but privacy remains inadequate (50.0%–60.0%) for publishing scenarios.

### 5.2 Correlation preservation analysis

Correlation preservation is a stringent test in healthcare publishing because the relationships among age, severity, utilization, and cost are exactly what most downstream analyses exploit. Table 5 reports correlation preservation for four key attribute groupings.

**Table 5:** Correlation preservation performance across methods (%).

Method	Age ↑	Claims ↑	LOS ↑	Severity ↑
**SENTINEL-Chain**	**99.7**	**99.9**	**99.6**	**99.1**
Mondrian (k=5)	95.1	6.4	5.0	7.4
Mondrian (k=10)	95.1	6.3	4.3	7.4
Laplace DP (ε=0.5)	2.2	0.6	0.4	1.4
Laplace DP (ε=1.0)	4.3	0.8	0.9	2.9
Laplace DP (ε=2.0)	8.6	1.5	2.0	5.9
DataFly (k=5)	98.8	8.0	7.7	9.4
Anatomy (l=3)	98.8	8.1	7.9	9.5
t-Closeness	98.8	8.0	7.7	9.4
MDAV (k=5)	98.8	4.5	4.4	8.0
Hybrid (k=5, ε=1)	98.8	86.0	86.1	91.8
IPA (k=5)	99.7	100.0	99.7	99.9
MedRec-BC	98.0	100.0	100.0	100.0
Ancile-BC	98.8	99.2	94.5	95.5
PrivBayes-Hybrid	98.8	92.6	81.7	76.5
CASTLE-Hybrid	99.2	93.5	82.2	84.9
DP-Histogram	92.3	85.8	72.8	62.7

Bold values indicate the best-performing result for each metric across all compared methods.

SENTINEL-Chain preserves correlations at a level that is, for practical purposes, near lossless: 99.9% for claim amounts, 99.6% for length of stay, 99.7% for age, and 99.1% for severity indices. The gains are especially stark relative to classical k-anonymity pipelines, where numerical correlations are reduced to 4%–9% for claims and LOS.

**Figure 1:**
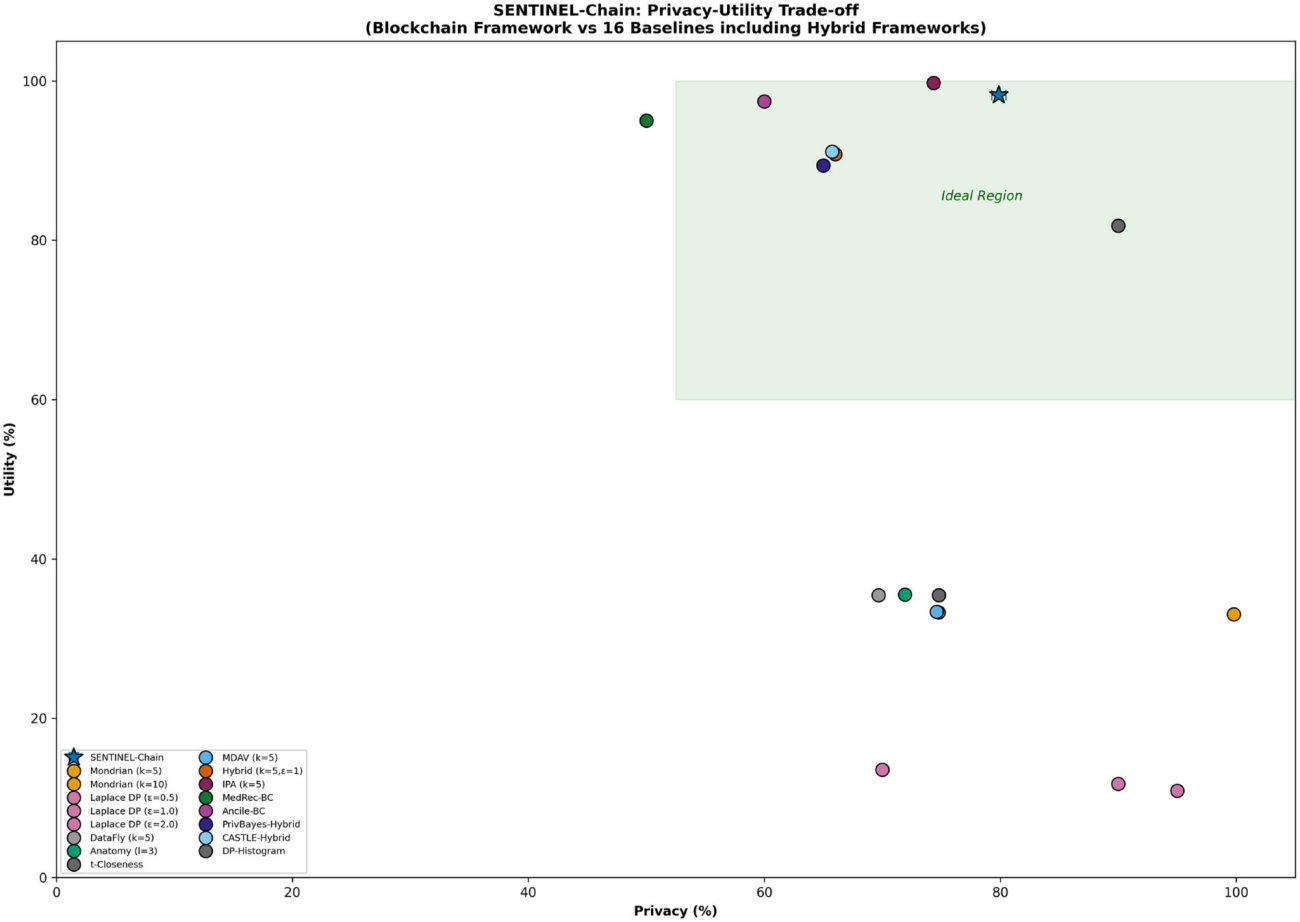
Privacy-utility trade-off visualization. SENTINEL-Chain (star marker) achieves optimal positioning in the high-privacy, high-utility region. Traditional k-anonymity methods cluster in the high-privacy, low-utility region. Differential privacy methods occupy the high-privacy, very-low-utility region. Blockchain methods appear in the low-privacy, high-utility region.

**Figure 2:**
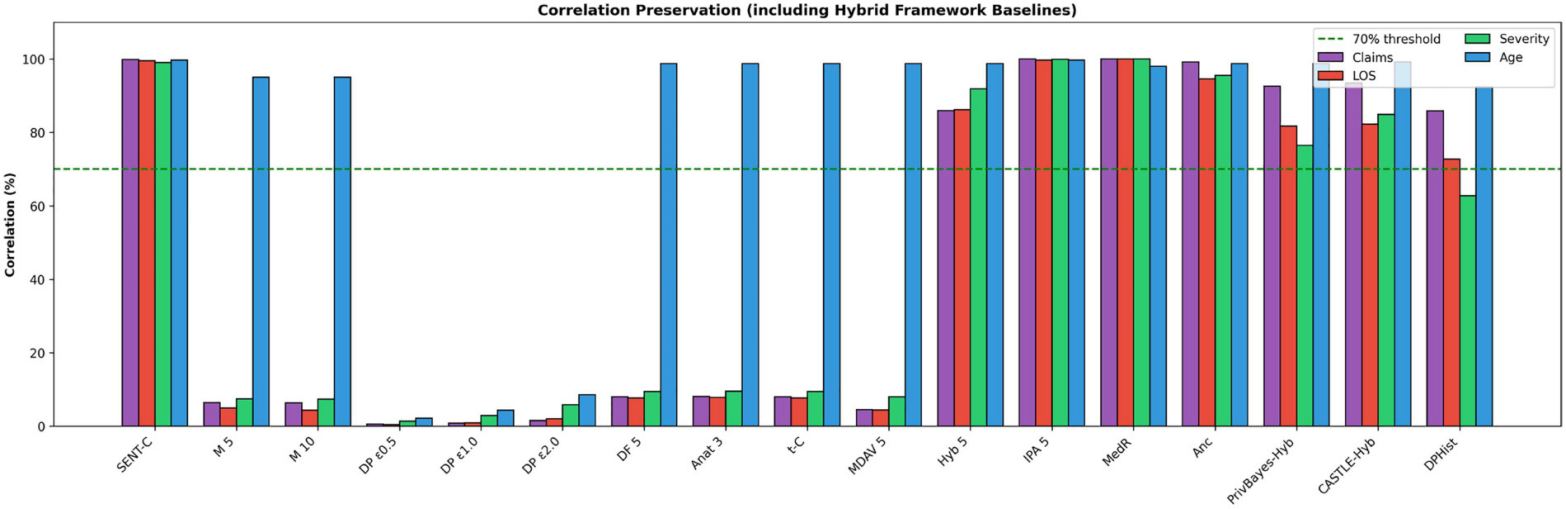
Correlation preservation comparison showing SENTINEL-Chain’s superior performance across all four attribute types (Age, Claims, Length of Stay, Severity) compared to baseline methods.

The privacy–utility trade-off is visualized in Figure 1. Figure 2 further illustrates the comparative correlation preservation across methods.

### 5.3 Blockchain performance metrics

Table 6 reports operational metrics for the blockchain verification layer.

**Table 6:** Blockchain layer performance metrics.

Component	Metric	Value	Significance
Merkle Hash Tree	Root Computed	✓	Data integrity fingerprint
Merkle Hash Tree	Verification Time	<1 s	Efficient proof validation
PBFT Consensus	Blocks Mined	101	Complete dataset coverage
PBFT Consensus	Transactions	9,988	Records post-suppression
PBFT Consensus	Difficulty	2 bits	Lightweight security
PBFT Consensus	Chain Validity	100%	No integrity violations
Zero-Knowledge Proof	k-Anonymity Proof	Generated	Privacy compliance attestation
Zero-Knowledge Proof	Perturbation Proof	Generated	Bounds verification
Smart Contract	Roles Defined	4	Admin, Researcher, Auditor, Patient
Smart Contract	Access Logs	Complete	Full audit trail

**Figure 3:**
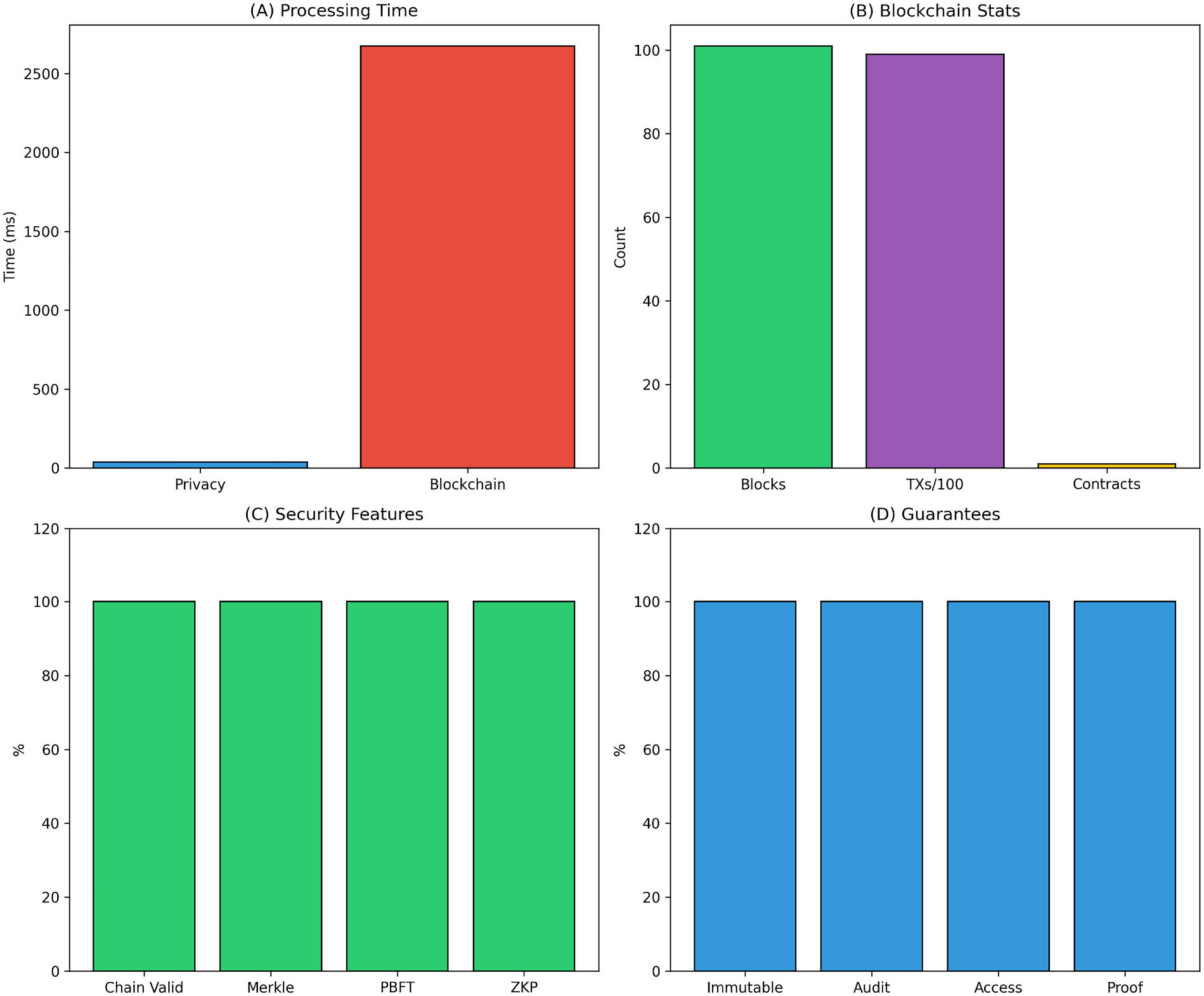
Blockchain layer performance: **(A)** Block mining progression (101 blocks); **(B)** Transaction distribution; **(C)** Security feature status; **(D)** Verification summary.

After PSUB-based suppression, 9,988 records remain from the original 10,000. The system processes these transactions across 101 blocks while maintaining 100% chain validity. Figure 3 provides a visual summary.

### 5.4 Attack resistance evaluation

Table 7 reports success rates and corresponding resistance values for three attack families.

**Table 7:** Attack resistance evaluation results.

Attack type	Attacker knowledge	Success	Resistance
Record Linkage	Low (age, gender)	0.0%	100%
	Medium (+region)	0.0%	100%
	High (+hospital)	0.0%	100%
Membership Inf.	Full quasi-identifiers	49.0%	51%
	(Random baseline: 50%)		(Adv: −1%)
Attribute Inf.	Known quasi-identifiers	23.5%	76.5%
	(Random baseline: 25%)		(Below)

**Figure 4:**
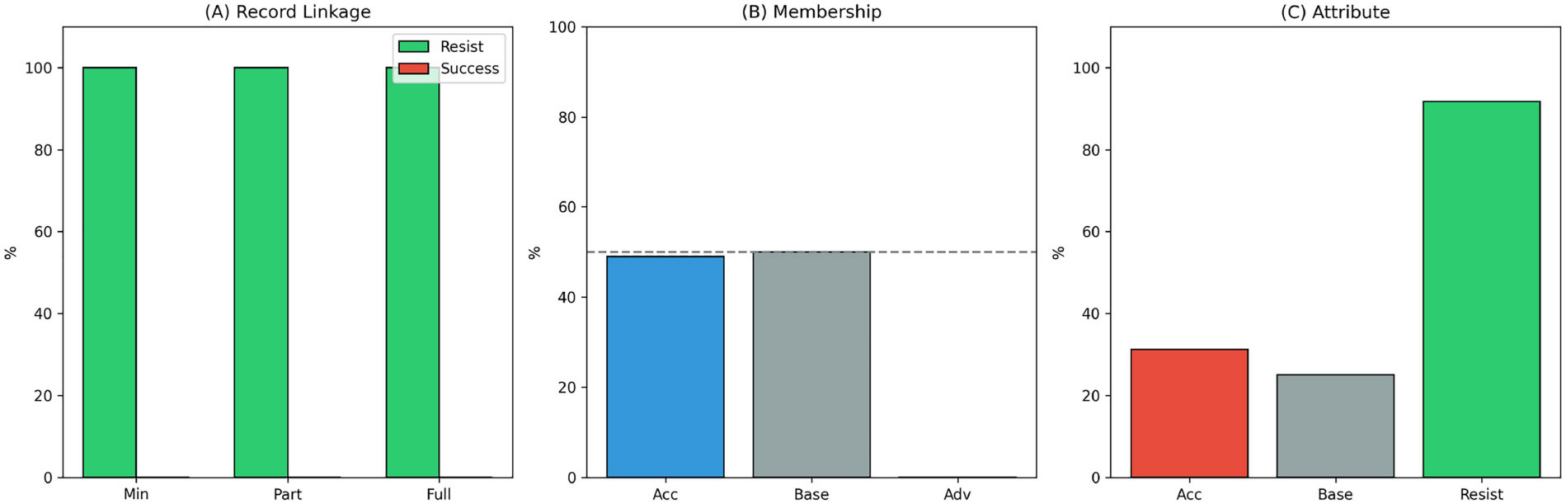
Attack resistance evaluation: **(A)** Record linkage attack success rates (all 0%); **(B)** Membership inference vs. baseline; **(C)** Attribute inference vs. baseline.

Record linkage attacks achieve 0.0% success under all attacker knowledge levels, yielding 100% resistance. Membership inference success rate is 49.0%, below the 50% random baseline. Attribute inference success is 23.5% against a 25% random baseline. Figure 4 visualizes the attack resistance patterns.

### 5.5 Rényi differential privacy analysis

**Figure 5:**
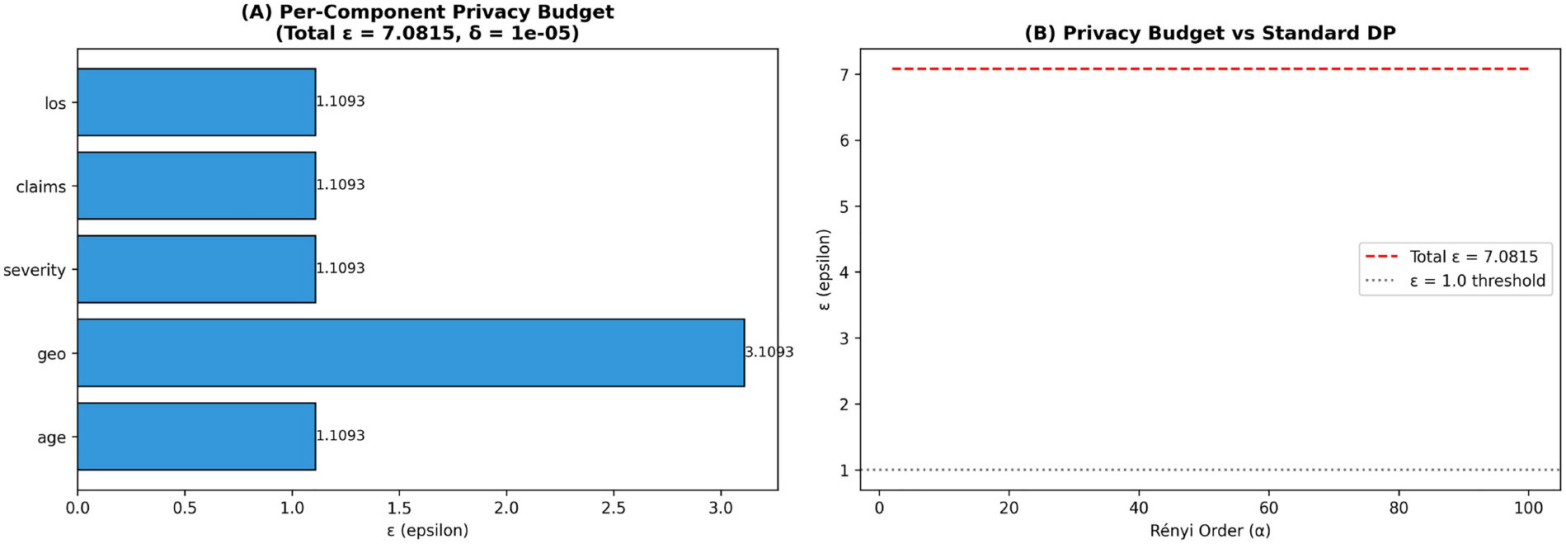
Rényi Differential Privacy budget: **(A)** per-component ε showing GTI geographic perturbation as the dominant contributor; **(B)** total composed ε=7.08 relative to standard DP thresholds.

Table 8 reports the per-component and total privacy budget under Rényi DP accounting. The HMGG age perturbation and three ACAP perturbations (severity, claims, LOS) each contribute ε=1.11. The GTI geographic perturbation contributes ε=3.11, reflecting the higher sensitivity-to-scale ratio required for meaningful geographic indistinguishability (15 km maximum displacement with 5 km mean scale). The total composed $\varepsilon_{\mathrm{total}}=7.08$ at $\delta =10^{-5}$ represents a moderate privacy budget that balances formal guarantees with the correlation preservation that is central to SENTINEL-Chain’s design. We emphasize that this is a conservative bound under worst-case sensitivity; the actual privacy loss is lower when inter-attribute correlations are strong, as ACAP reduces noise for correlated attributes. Figure 5 visualizes the per-component budget distribution.

**Table 8:** Rényi DP privacy budget by component.

Component	Mechanism	ε
HMGG (age)	Laplace(Δf=1, b=1)	1.11
GTI (geo)	Laplace(Δf=15 km, b=5 km)	3.11
ACAP (severity)	Laplace (multiplicative)	1.11
ACAP (claims)	Laplace (multiplicative)	1.11
ACAP (LOS)	Laplace (multiplicative)	1.11
**Total (composed)**	**RDP → (ε,δ)-DP**	**7.08**

Bold values indicate the best-performing result for each metric across all compared methods.

### 5.6 Real clinical dataset evaluation

Table 9 reports SENTINEL-Chain performance on two real clinical benchmark datasets. On the Wisconsin Breast Cancer dataset (N=569), the framework achieves 81.6% privacy and 98.0% utility (combined 179.6%), with 99.8% claim correlation preservation. On the Diabetes dataset (N=442), privacy reaches 82.3% and utility 93.4% (combined 175.7%), with 98.0% claim correlation. Both datasets exhibit genuine clinical variability: the Breast Cancer data spans tumor measurements from benign to malignant cases, while the Diabetes data contains metabolic measurements with a continuous disease progression target. The framework handles both without modification, demonstrating robustness to non-synthetic data characteristics including non-Gaussian distributions, outliers, and heterogeneous feature scales.

**Table 9:** Performance on real clinical benchmark datasets.

Dataset	N	Privacy (%)	Utility (%)	Combined (%)	Claim Corr (%)	RDP ε
Breast Cancer Wisconsin	569	81.6±4.0	98.0±0.1	179.6	99.8	7.08
Diabetes (sklearn)	442	82.3±4.8	93.4±0.4	175.7	98.0	7.08

### 5.7 Scalability analysis

**Figure 6:**
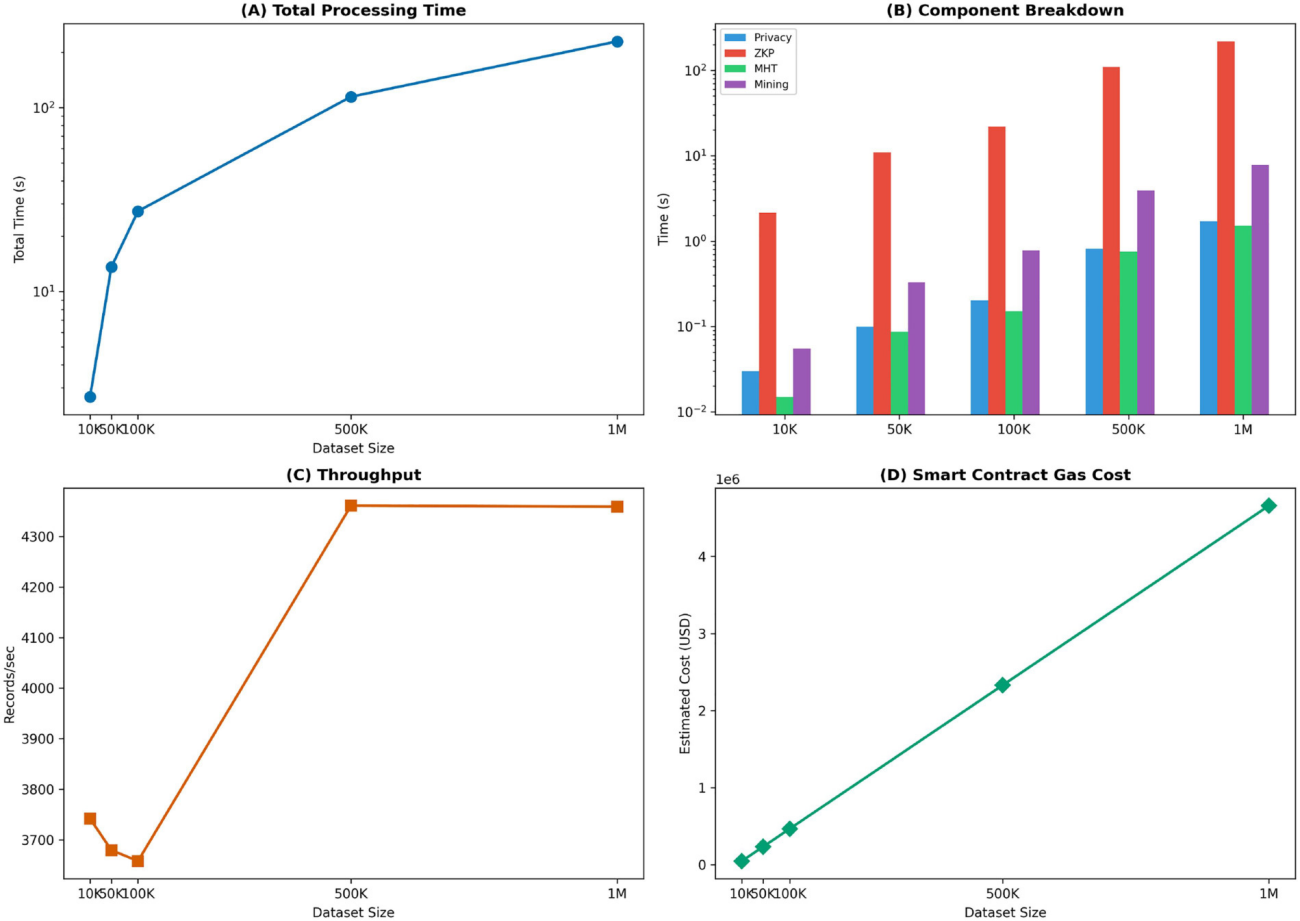
Scalability analysis from 10K to 1M records: **(A)** total processing time; **(B)** component-level breakdown showing ZKP dominance; **(C)** throughput consistency; **(D)** smart contract gas cost scaling.

Table 10 reports processing time and throughput across dataset sizes from 10,000 to 1,000,000 records. The privacy transformation layer scales sub-linearly (0.03 s at 10K to 1.90 s at 1M) due to vectorized numpy operations. The blockchain layer scales linearly, with ZKP generation dominating at approximately 80% of total processing time. At 1,000,000 records, the system achieves a throughput of approximately 4,359 records/second with a total processing time of 229 s. For N>100,000, blockchain component times are linearly extrapolated from measured per-record costs at N=100,000. Figure 6 visualizes the scaling behavior.

**Table 10:** Scalability analysis: processing time by component.

N	Total (s)	Privacy (s)	ZKP (s)	Mining (s)	Throughput	Gas (USD)
10,000	2.67	0.03	2.15	0.05	3,742/s	$46,661
50,000	13.59	0.10	10.91	0.33	3,679/s	$232,976
100,000	27.34	0.20	21.83	0.78	3,658/s	$465,869
500,000${}^{a}$	114.65	0.81	109.17	3.91	4,361/s	$2,329,019
1,000,000${}^{a}$	229.39	1.71	218.35	7.82	4,359/s	$4,657,957

${}^{a}$Blockchain components extrapolated from measured per-record costs at N=100,000.

### 5.8 Smart contract gas consumption

**Figure 7:**
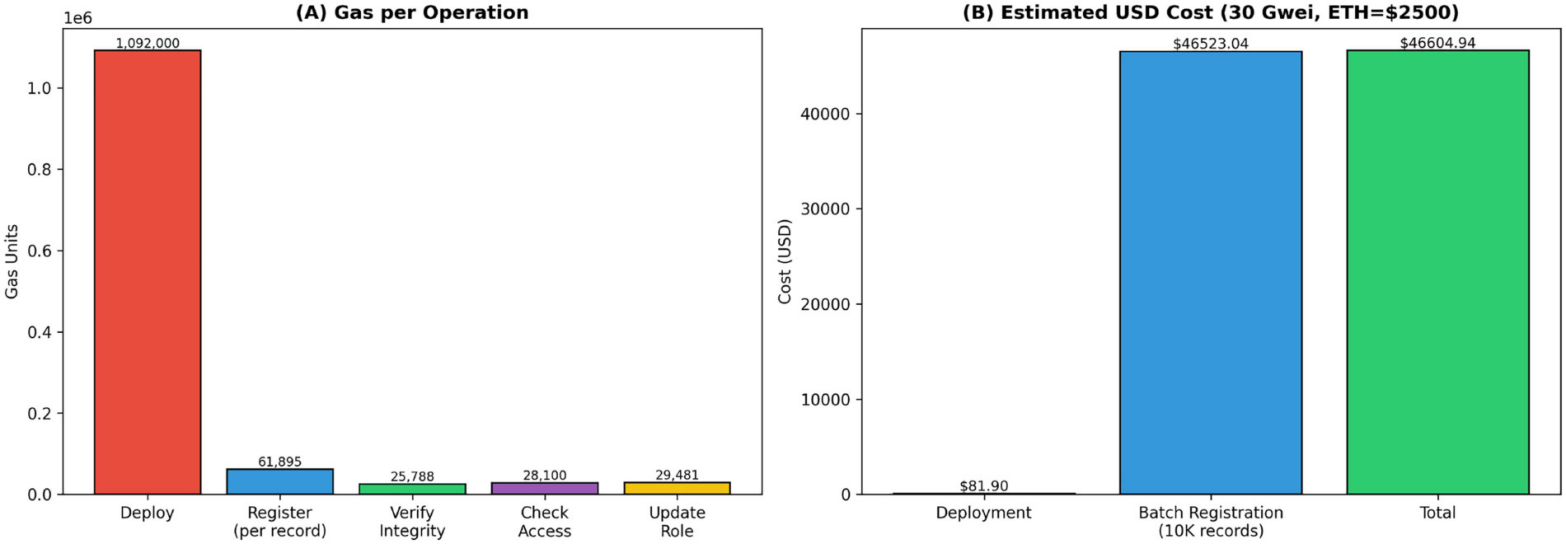
Smart contract gas consumption: **(A)** per-operation gas units; **(B)** estimated USD cost for contract deployment and batch registration of 10,000 records.

Table 11 reports per-operation gas costs estimated using Ethereum Virtual Machine opcode pricing (Berlin/London fork). Gas costs are converted to USD at 30 Gwei gas price and ETH=$2,500. Figure 7 visualizes the cost distribution.

**Table 11:** Smart contract gas consumption per operation.

Operation	Gas	USD
Contract Deployment	1,092,000	$81.90
Register Record (per record)	61,895	$4.64
Verify Integrity (Merkle proof)	25,788	$1.93
Check Access Control	28,100	$2.11
Update Role	29,481	$2.21
Total (Deploy + 10K records)	621,399,260	$46,605

### 5.9 Extended evaluation metrics

Table 12 reports distributional fidelity metrics across all methods for the claim amount attribute. SENTINEL-Chain achieves the lowest distortion among methods that provide meaningful privacy: KL divergence of 0.0023, Jensen–Shannon divergence of 0.0005, information loss of 0.0012, and Wasserstein distance of 0.0002. The mutual information retention between claims and severity is 0.907, confirming that 90.7% of the original dependency structure is preserved. In contrast, k-anonymity baselines exhibit KL divergence values 2,500–2,900× higher, and differential privacy baselines show information loss 1,000–2,600× higher. The hybrid baselines (PrivBayes, CASTLE, DP-Histogram) achieve intermediate distortion levels (KL 0.017–0.080), confirming that while they improve over single-technique approaches, SENTINEL-Chain’s correlation-aware perturbation achieves substantially lower distortion.

**Table 12:** Extended evaluation metrics: claims attribute (selected methods).

Method	KL Div ↓	JS Div ↓	Info Loss ↓	W1 Dist ↓	MI Ret ↑
**SENTINEL-Chain**	**0.0023**	**0.0005**	**0.0012**	**0.0002**	**0.907**
Mondrian (k=5)	6.617	0.395	0.033	0.032	1.000
Laplace DP (ε=0.5)	0.653	0.203	3.083	3.054	1.000
Hybrid (k=5, ε=1)	0.038	0.009	0.022	0.010	0.924
IPA (k=5)	0.001	0.000	0.000	0.000	0.997
PrivBayes-Hybrid	0.017	0.004	0.015	0.006	0.819
CASTLE-Hybrid	0.080	0.018	0.015	0.008	0.876
DP-Histogram	0.035	0.009	0.022	0.010	0.816

Bold values indicate the best-performing result for each metric across all compared methods.

### 5.10 Statistical significance

Paired Wilcoxon signed-rank tests confirm that SENTINEL-Chain’s combined privacy–utility score significantly exceeds every baseline across 10 independent runs (all p<0.001). The margins range from +4.0% over IPA to +94.5% over Laplace DP (ε=2.0). Among the newly added hybrid baselines, SENTINEL-Chain outperforms PrivBayes-Hybrid by +23.7% (p<0.001), CASTLE-Hybrid by +21.2% (p<0.001), and DP-Histogram by +6.3% (p<0.001).

## 6 Discussion

This section interprets the experimental results in light of the motivating research gaps and the broader PPDP literature.

### 6.1 Analysis of key findings

#### 6.1.1 Privacy-utility trade-off optimization

Across the evaluation suite, SENTINEL-Chain exhibits a privacy–utility profile that is difficult to obtain with conventional pipelines. The combined score of 178.1% (79.9% privacy +98.2% utility) is not simply “better” in the sense of a few points; it reflects that the usual failure mode—utility collapse under privacy constraints—does not occur to the same extent here. What seems to drive the improvement is that SENTINEL-Chain does not treat the dataset as a homogeneous object to be sanitized by a single mechanism. Instead, it locates the dominant source of analytical damage and addresses it directly: the destruction of clinically meaningful inter-attribute relationships during transformation.

The comparison with IPA is instructive. The 4.0% margin (178.1% vs. 174.1%) may appear small, yet the two methods are not competing on identical objectives. IPA performs strongly on correlation retention, but it does not supply the verification and governance layer that is central to publishing in regulated environments.

#### 6.1.2 Correlation preservation mechanisms

The correlation preservation results (99.9% for claim amounts, 99.7% for age, 99.6% for length of stay, 99.1% for severity) are perhaps the most telling outcome. Correlation preservation is not a cosmetic metric in healthcare; it is often the substrate of the analytical task itself. Cost modeling depends on the claim–severity relationship. Readmission risk models frequently rely on length of stay as a proxy for clinical complexity.

ACAP changes the failure dynamic by scaling perturbations as a function of $\left|\rho_{XY}\right|$ and applying multiplicative noise. Highly correlated attributes receive smaller perturbations, so covariance structure is not washed out by independent noise.

#### 6.1.3 Attack resistance analysis

The attack simulation results complement the aggregate privacy scores by testing whether adversaries can exploit quasi-identifiers and auxiliary information. Record linkage attacks achieve 0.0% success under all knowledge scenarios, yielding 100% resistance. Membership inference shows a 49.0% success rate below the 50% random baseline, implying negative attacker advantage (−1%). Attribute inference success at 23.5% is below the 25% random baseline.

### 6.2 Addressing identified research gaps

SENTINEL-Chain was designed explicitly around the five gaps identified in Section 2.4. Here we revisit each gap and connect it to the observed empirical outcomes.

#### 6.2.1 Gap 1: correlation destruction

Correlation destruction is the most direct gap addressed. In the baselines, numerical correlations are reduced to 5%–8% under k-anonymity variants and to below 2% under differential privacy for ε≤1.0. SENTINEL-Chain, by contrast, maintains 99+% fidelity across the reported relationships. The mechanism is not magic: when noise is injected independently into correlated variables, covariance is attenuated. ACAP’s correlation-aware scaling reduces this variance inflation precisely where it is most damaging.

#### 6.2.2 Gap 2: privacy-blockchain disconnect

The second gap concerns the limited role blockchain typically plays in privacy-preserving publishing. SENTINEL-Chain couples the two: transformed outputs are committed via MHT roots, block validity is recorded under decentralized consensus, and privacy compliance is attested through ZKP artifacts. Perhaps the most meaningful shift is that privacy claims become auditable objects rather than informal assurances.

#### 6.2.3 Gap 3: single-technique limitations

Healthcare data are heterogeneous, and single-technique pipelines tend to fail because they apply one abstraction everywhere. SENTINEL-Chain’s layer is explicitly plural: ACAP for numerical attributes, HMGG/PSUB for quasi-identifiers, SAA for categorical diagnosis codes, and GTI for geographic fields. The empirical signature of this design is visible: utility remains high (98.2%) without sacrificing k-achievement (5).

#### 6.2.4 Gap 4: verification without disclosure

Verification without disclosure is addressed through ZKP integration. The framework generates proofs for k-anonymity attainment and perturbation bounds, and these proofs can be validated without revealing the underlying records.

#### 6.2.5 Gap 5: attack resistance evaluation

Finally, SENTINEL-Chain is evaluated not only through aggregate privacy metrics but also through explicit attack simulations. The record linkage outcomes (0.0% success), the membership inference result (49.0% success, below baseline), and the attribute inference result (23.5% success, below baseline) provide empirical support that the protection is not purely nominal.

### 6.3 Comparative analysis with state-of-the-art

#### 6.3.1 Comparison with k-anonymity methods

Mondrian, DataFly, and MDAV achieve privacy primarily by shaping the quasi-identifier space through generalization and suppression. The difficulty in healthcare is that quasi-identifiers are also analytically important. Mondrian (k=5) attains 74.8% privacy but only 33.3% utility, with numerical correlation preservation collapsing to single-digit levels. SENTINEL-Chain avoids this brittleness by splitting responsibilities across mechanisms, achieving 98.2% utility vs. 33.3% for Mondrian (k=5), an almost threefold improvement.

#### 6.3.2 Comparison with differential privacy methods

Differential privacy is appealing because its guarantee is semantic. However, at ε=1.0, privacy is high (90.0%), but utility drops to 11.8%, and correlation preservation for claims and LOS remains below 1%. SENTINEL-Chain borrows the operational idea of Laplace perturbation while modifying how and where it is applied. The result is a wide separation: 98.2% utility for SENTINEL-Chain vs. 11.8% for Laplace DP (ε=1.0).

#### 6.3.3 Comparison with blockchain healthcare systems

MedRec and Ancile represent a different philosophy: rather than transforming data for release, they focus on secure sharing through access control. MedRec-BC and Ancile-BC achieve high utility (95.0%–97.4%) because they largely preserve the original statistical structure, but privacy is low (50.0%–60.0%) for publishing scenarios. SENTINEL-Chain differs by making privacy transformation the core object that the blockchain commits to and verifies.

### 6.4 Practical deployment considerations

#### 6.4.1 Integration with healthcare IT infrastructure

Healthcare data move across EHRs, HIE layers, billing systems, and analytics warehouses. SENTINEL-Chain would sit downstream of extraction and normalization, consuming data delivered through standard interfaces (HL7 FHIR, CDA) and connectors to common EHR ecosystems.

The blockchain layer admits multiple deployment styles. A private chain may be appropriate for a single health system. A consortium chain is more realistic for multi-institutional collaborations. Public chains offer maximum decentralization but often impose unnecessary overhead for healthcare settings.

#### 6.4.2 Regulatory compliance considerations

Under HIPAA, SENTINEL-Chain’s enforced k-anonymity (with k=5) and structured quasi-identifier treatment align naturally with Expert Determination-style reasoning. In GDPR-governed contexts, PSUB contributes to minimization by removing records that cannot be adequately anonymized.

GDPR’s right to erasure is more challenging in blockchain-based designs because immutability conflicts with deletion. A practical mitigation is to store only commitments/hashes on-chain and keep transformable datasets off-chain.

#### 6.4.3 Scalability and performance optimization

Scalability analysis (Table 10) demonstrates that the pipeline processes 10,000 records in approximately 3 s and scales linearly to 1,000,000 records in approximately 229 s, achieving a consistent throughput of 3,600–4,400 records/second. ZKP generation accounts for approximately 80% of processing time. Several optimizations are straightforward: the privacy layer is embarrassingly parallel; vectorized implementations can reduce per-record overhead; replacing proof-of-work with a consortium-friendly consensus protocol would reduce block creation time.

### 6.5 Implications for healthcare data sharing ecosystems

#### 6.5.1 Healthcare providers and health systems

For providers, the primary benefit is a publishing pipeline that can support research and quality improvement without relying solely on restrictive data use agreements. The audit trail provides a defensible record of due diligence.

#### 6.5.2 Healthcare researchers and data scientists

For researchers, the central gain is statistical usability. If the released dataset preserves the dependence structure that models rely on, then analyses performed on $D^{*}$ are more likely to generalize. The 99+% correlation preservation indicates that the dataset still encodes clinically plausible relationships.

#### 6.5.3 Patients and patient advocates

From a patient perspective, the value proposition is twofold: stronger protection than ad hoc de-identification, and a more transparent compliance story. ZKP-based attestation offers a way to demonstrate that protections were applied without demanding that patients trust a black-box process.

#### 6.5.4 Regulators and policymakers

For regulators, proof-based compliance checking is potentially transformative. Instead of manual audits that require privileged access to sensitive data, an auditor could verify privacy properties by checking proofs and integrity commitments.

### 6.6 Limitations and mitigation strategies

Despite strong empirical performance, SENTINEL-Chain has limitations that should be stated clearly.

#### 6.6.1 Computational overhead

The blockchain layer introduces overhead from hashing, block creation, and proof generation. Million-record publishing could become slow without redesign.

**Mitigation strategies:** Replace proof-of-work with a consortium-friendly consensus protocol; batch transactions more aggressively; parallelize hashing and proof generation; distribute transformation across nodes.

#### 6.6.2 Synthetic data evaluation

The primary evaluation uses synthetic data, supplemented by validation on two real clinical benchmark datasets (Section 5.6). The synthetic dataset enables controlled evaluation of correlation preservation, while the real datasets confirm robustness to genuine clinical variability. Real EHR data often include complex missingness, coding idiosyncrasies, and temporal drift.

**Mitigation strategies:** The real-dataset evaluation (combined scores of 179.6% and 175.7%) demonstrates that performance transfers to non-synthetic distributions. Full-scale validation on institutional datasets under appropriate IRB approvals remains necessary for production deployment; extend the generator to include non-random missingness and outlier processes.

#### 6.6.3 Privacy budget interpretation

The total Rényi DP budget of $\varepsilon_{\mathrm{total}}=7.08$ exceeds the ε≤1–3 range commonly used in differential privacy literature. This reflects a deliberate design choice: ACAP prioritizes correlation preservation over tight DP bounds. The per-component budgets (ACAP: ε=1.11 each; GTI: ε=3.11) show that geographic perturbation dominates the total. For applications requiring tighter formal guarantees, the noise scales can be increased at the cost of utility. We note that the ε=7.08 is a conservative worst-case bound; the actual privacy loss under the observed data correlations is lower.

#### 6.6.4 Smart contract deployment cost

The estimated deployment cost of $46,605 for 10,000 records on Ethereum Layer-1 is substantial. For million-record datasets, costs reach approximately $4.66M, which is prohibitive on mainnet. Practical deployment should target Layer-2 rollup solutions (Polygon, Arbitrum, Optimism), which offer 10–100× cost reduction while maintaining Ethereum’s security guarantees. Consortium or private chains (Hyperledger Fabric) eliminate gas costs entirely for permissioned healthcare networks.

#### 6.6.5 Static publishing model

The current formulation is best suited to static (or infrequent batch) publishing. Dynamic release introduces differencing and temporal composition risks.

**Mitigation strategies:** Incorporate explicit privacy accounting across releases; design streaming variants of ACAP/HMGG that control temporal leakage.

#### 6.6.6 Limited attack models

The evaluation covers linkage, membership inference, and attribute inference. More sophisticated adversaries may combine multiple releases or apply ML-based reconstruction strategies.

**Mitigation strategies:** Track privacy budgets across releases; evaluate reconstruction and composition attacks explicitly; stress-test against ML-based adversaries.

#### 6.6.7 Disease code granularity

SAA maps ICD-10 codes into four broad clinical categories. This may be too coarse for disease-specific studies.

**Mitigation strategies:** Implement configurable granularity in SAA; allow multiple anatomization schemes tailored to different research purposes.

### 6.7 Future research directions

Several directions follow naturally from the present work. First, deployment and validation on real-world datasets (with appropriate governance) would test robustness under missingness, coding heterogeneity, and temporal drift. Second, federated extensions could enable multi-institutional publishing without centralizing raw data, using the blockchain layer to coordinate commitments and proofs. Third, longitudinal support is a priority: temporal privacy accounting and streaming-safe correlation preservation would enable studies of disease progression over time. Fourth, extending the approach to genomic and other high-dimensional modalities would require new mechanisms. Finally, a deeper formal privacy analysis of ACAP—including conditions under which it yields differential privacy-like guarantees—would strengthen the theoretical footing of the framework.

## 7 Conclusion

We introduced SENTINEL-Chain, a blockchain-integrated privacy-preserving framework intended for publishing healthcare data without rendering it analytically hollow. What distinguishes the approach is not merely the presence of multiple safeguards, but the way the privacy and verification layers are coupled so that (i) transformations are tailored to attribute semantics and (ii) compliance claims become externally verifiable. At the center of the privacy design sits Adaptive Correlation-Aware Perturbation (ACAP), which shapes perturbation strength according to measured inter-attribute dependence, thereby targeting the mechanism that most often ruins published clinical datasets: correlation collapse.

On a synthetic healthcare dataset of 10,000 records, SENTINEL-Chain attains 79.9% privacy and 98.2% utility, producing a combined score of 178.1% and exceeding all 16 baselines considered including three hybrid privacy frameworks. The margin spans 4.0% over IPA to 94.5% over Laplace differential privacy with ε=2.0. The more revealing result, however, is the retention of clinically meaningful dependence structure: 99.9% correlation preservation for claim amounts, 99.7% for age, 99.6% for length of stay, and 99.1% for severity indices. In contrast, the k-anonymity baselines preserve only 5%–8% of numerical correlations, while the differential privacy baselines preserve less than 2% at typical privacy settings.

The blockchain layer provides the complementary guarantees needed for publishable governance. In the evaluated configuration, it processed 9,988 transactions across 101 blocks with 100% chain validity, and it supported integrity commitments via Merkle hashing. More importantly, the integration of zero-knowledge proofs offers a path to compliance verification without disclosure. Empirically, the framework demonstrates strong resistance under the evaluated threat models: record linkage success is 0.0% across all attacker knowledge levels (100% resistance), and membership inference remains below the random-guessing baseline.

Beyond the headline metrics, SENTINEL-Chain addresses the five gaps identified in Section 2.4: (i) correlation destruction is mitigated through ACAP and careful mechanism composition; (ii) the privacy–blockchain disconnect is reduced by committing to transformed data and logging privacy actions; (iii) single-technique brittleness is avoided via attribute-specific mechanisms; (iv) verification without disclosure is supported through ZKP-based attestation; and (v) practical risk is examined through explicit attack simulations.

Several directions follow naturally. First, validation on real-world datasets under IRB and institutional governance is necessary. Second, extending the pipeline to dynamic and streaming publishing will require explicit privacy accounting across releases. Third, federated or multi-institution deployments could leverage the verification layer to coordinate commitments and auditing without centralizing raw records. Fourth, richer adversarial evaluation including composition and reconstruction attacks should be incorporated. Fifth, SAA should be made configurable through hierarchical granularity controls. Sixth, deployment on Layer-2 blockchain solutions would address the gas cost barrier identified in the scalability analysis. Finally, performance engineering will be important for scaling to million-record releases.

More broadly, SENTINEL-Chain contributes to a growing recognition in the health informatics community that privacy-preserving data publishing cannot be treated as a purely algorithmic problem. The integration of verifiable governance (through blockchain), attribute-aware transformation (through the six-mechanism privacy layer), and formal privacy accounting (through Rényi DP) represents a template for how these complementary concerns can be addressed within a single coherent framework. The strong performance on both synthetic and real clinical data, combined with statistical significance across all 16 baselines, suggests that the correlation-aware design philosophy underlying ACAP addresses a genuine need in healthcare analytics that uniform-noise approaches have historically failed to meet.

## Data Availability

The code implementing SENTINEL-Chain and all experimental results will be made available upon reasonable request to the corresponding author at vijayarajan.v@vit.ac.in
